# Effects of Different Whitening Toothpastes on the Color of Aged Zirconia‐Reinforced Lithium Silicate Ceramics After Immersion in an Acidic Beverage

**DOI:** 10.1155/bmri/6451392

**Published:** 2026-09-06

**Authors:** Ali Reza Akbarnia, Elham Ahmadi, Ladan Ranjbar Omrani, Amir Mohammad Azizzadeh, Ahmad Reza Shamshiri, Fatemeh Farshad, Mahdi Abbasi

**Affiliations:** ^1^ Department of Restorative Dentistry, School of Dentistry of Tehran University of Medical Sciences, Tehran, Iran; ^2^ Department of Environmental Health Engineering, School of Public Health of Tehran University of Medical Sciences, Tehran, Iran; ^3^ Dental Research Center, Dentistry Research Institute of School of Dentistry of Tehran University of Medical Sciences, Tehran, Iran

**Keywords:** ceramics, cola, color, toothpastes

## Abstract

**Introduction:**

This study is aimed at comparing the effects of different whitening toothpastes on the color of aged ZLS ceramics after immersion in an acidic beverage.

**Materials and Methods:**

In this in vitro study, 72 cylindrical (2×10 mm) ZLS ceramic specimens were sintered, polished, and randomly assigned to six groups (*n* = 12) of distilled water (D), distilled water + Colgate Total Whitening (DT), distilled water + Perfect White black (DP), cola (C), cola + Colgate Total Whitening (CT), and cola + Perfect White black (CP). After 5000 thermal cycles, the specimens were immersed in an acidic beverage for 6 days. The specimens in groups CP, CT, DP, and DT were brushed at 40 rpm for 2.33 min. The CIEL ^∗^a ^∗^b ^∗^ color parameters were measured spectrophotometrically before and after aging and toothbrushing. Data were analyzed by one‐way ANOVA (alpha = 0.05).

**Results:**

The color change (*Δ* *E*) was not clinically detectable in any group (< 3.3). *Δ* *E* of the CT group was significantly greater than that of the C (mean difference: 0.12, *p* < 0.05) and the CP (mean difference: 0.13, *p* < 0.05) groups. *Δ* *L* of the C group was significantly greater than that of the CP group (mean difference: 0.21, *p* < 0.05). The differences in *Δ* *a* and *Δ* *b* were not significant among the groups (*p* > 0.05).

**Conclusions:**

The application of Colgate Total Whitening and Perfect White black toothpaste for 6 weeks, along with simulated 6‐month cola consumption, did not cause a clinically detectable change in the color of aged ZLS ceramics.

## 1. Introduction

All‐ceramic restorations are increasingly used for their optimal visual and optical properties, comparable to those of natural teeth [1, 2]. Dental ceramic restorations are fabricated by various methods, including slip casting, the lost‐wax/heat‐pressed technique, powder condensation, and computer‐aided design and computer‐aided manufacturing (CAD/CAM) technology [3]. The CAD/CAM method is cost‐effective compared with other conventional methods and provides acceptable color and contour, as well as favorable marginal fit and shade matching. The monolithic CAD blocks are now available with a lower fracture rate than handmade veneers [4]. Among monolithic CAD blocks, lithium disilicate ceramic blocks have gained popularity because of their versatility and practicality in fabricating indirect restorations, as well as their potential for postmilling staining [5]. Although this group of ceramics has a high application in dentistry, efforts to improve their properties and mechanical strength have led to the introduction of nanocrystalline ZrO_2_‐SiO_2_ glass‐ceramics, with nanozirconium oxide particles uniformly embedded in an amorphous SiO_2_ matrix. The addition of zirconia to this group of glass ceramics has made this material an excellent choice for ceramic veneers [6]. On the other hand, increased strength is another important factor contributing to the increasing popularity of this group of glass ceramics. Monolithic blocks of zirconia‐reinforced lithium silicate (ZLS) ceramic (VITA Suprinity; Vita, Bad Sackingen, Zahnfabrik, Germany) are currently used to make inlays, onlays, and veneers in the anterior and premolar regions [3, 7].

Although ceramics have excellent mechanical properties, they are subject to staining and changes in microhardness and surface roughness in the dynamic environment of the oral cavity [8]. Consumption of colored beverages and acidic solutions, temperature rise, and toothbrushing are among the external factors that can change the properties of ceramics [9].

Absorption of exogenous pigments in the oral cavity depends on the composition and surface topography of ceramics [5]. Lower porosity in the ceramic surface results in greater color stability. It means that machined ceramics (CAM) show better color stability than handmade laboratory‐fabricated types [10]. A previous study on the color properties and surface roughness of ZLS showed that the ceramics were affected by 10,000 thermal cycles, and that ZLS and feldspathic glass ceramics were more susceptible to aging than other ceramics [11]. Also, an investigation into the effects of finishing and aging processes on the surface roughness and color stability of ZLS ceramics was conducted, and it was found that 5000 thermal cycles significantly increased surface roughness but did not significantly change color [12].

Moreover, the effects of acidic environments such as acidic beverages [13] and simulated gastric acid [14] on properties such as microhardness, surface roughness, and color of ZLS ceramics have been studied previously. However, there have been a limited number of studies on the effect of whitening toothpastes on ZLS ceramics. A previous study [15] examined the effect of charcoal whitening toothpaste on color stability and gloss of zirconia and lithium disilicate ceramic blocks over 6 months, 1 year, and 6 years, and showed a significant reduction in gloss within 6 months and the occurrence of color change (*Δ* *E*) within 3 and 6 years in all ceramics. Walsh et al. [16] reported that abrasive whitening toothpastes resulted in clinically significant reductions in staining and improved whitening efficacy within 6 weeks. Therefore, the routine recommendation of 6 weeks of use of whitening toothpastes for maximum efficacy with minimal adverse effects on the oral environment appears reasonable in principle.

Because the oral environment is dynamic and changes in temperature, acidity, and abrasiveness occur simultaneously, however, previous studies have not considered the combined effect of these parameters, as they might have a synergistic effect in terms of color change. The present study is aimed at investigating the effect of different whitening toothpastes on the color of aged ZLS ceramics after immersion in an acidic beverage.

## 2. Methodology

This in‐vitro experimental study was conducted on ZLS ceramic specimens (A2‐H2; Vita Suprinity, VITA Zahnfabrik, Bad Sackingen, Germany). The study protocol was approved by the university ethics committee (IR.TUMS.DENTISTRY.REC.1399.206).

### 2.1. Sample Size

The sample size was calculated using the PASS 11 software (Number Cruncher Statistical Systems, LLC, United States) in the analysis of variance section of the fixed effect menu. The minimum required sample size was calculated to be eight specimens per group (Alpha = 0.05, Statistical Power = 0.2). However, because of the risk of specimen loss during the experiment, 12 specimens were included in each group.

### 2.2. Specimen Preparation

The blocks were cut into 72 cylindrical specimens with a 10 mm diameter and 2 mm thickness by using a jewelry cutter. Then, the specimens were sintered according to the manufacturer′s instructions. They were heated at 400°C for 4 min, then heated at 55°C/min to 840°C over 8 min. After 8 min of firing at this temperature, the ceramic blocks were placed in a vacuum machine until the temperature decreased to 680°C. Next, the specimens were polished. One of the two surfaces of each specimen was marked with a round bur and a high‐speed handpiece, and the opposite surface was used in this study.

**Figure 1:**
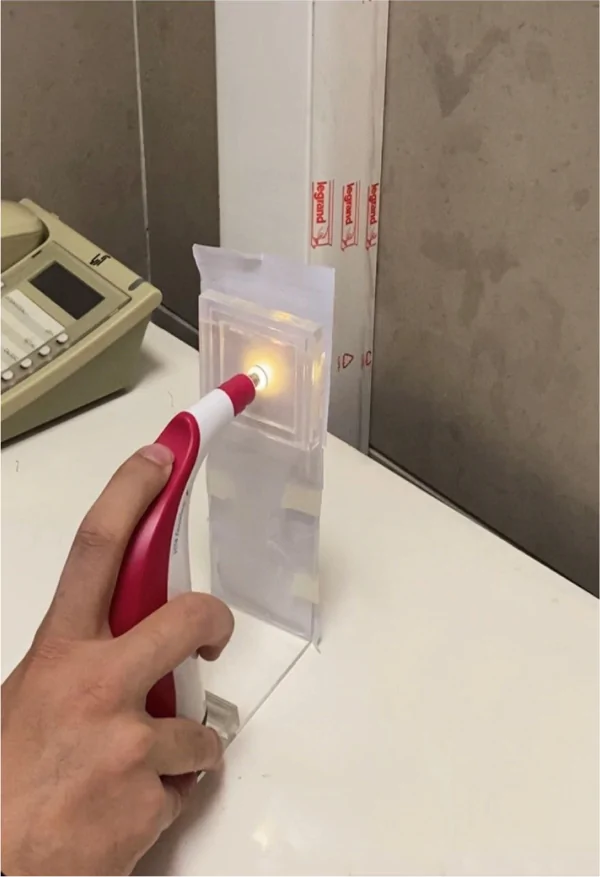
Device designed to ensure reproducible tooth positioning for color measurement.

The initial color of the specimens was determined by a spectrophotometer (Easyshade; VITA Zahnfabrik, Bad Sackingen, Germany) using the CIE L ^∗^a ^∗^b ^∗^ color system. To standardize color assessment conditions, a device was designed with transparent sheets consisting of a horizontal and a vertical plate; the horizontal plate was for positioning the spectrophotometer, and the vertical plate had an 11 mm diameter hole for placing the specimens. The spectrophotometer was placed in a fixed position perpendicular to the specimen surface, and a white plate was placed in the background (Figure 1). Before each color assessment, the spectrophotometer was calibrated according to the manufacturer′s instructions. The L ^∗^ (luminance), a ^∗^ (color on the green‐red axis), and b ^∗^ (color on the blue‐yellow axis) color parameters were measured for each specimen three times, and the mean values were recorded.

Two types of toothpastes were used for toothbrushing in this study, the composition of which is presented in Table 1.

**Table 1:** Composition of the toothpastes used.

Toothpaste	Composition
Perfect White black (Beverly Hills Formula, Ireland)	Water, sorbitol, hydrated silica, glycerin, pentasodium triphosphate, tetrasodium pyrophosphate, sodium lauryl sulfate, aroma, PEG‐32, cellulose gum, sodium fluoride, cocamidopropyl betaine, sodium saccharin, charcoal power and limonene
Colgate Total Whitening (ColgatePalmolive Company, New York, New York, United States)	Water, hydrated silica, glycerin, sorbitol, PVM/MA copolymer, sodium lauryl sulfate, flavor, cellulose gum, sodium hydroxide, propylene glycol, carrageenan, sodium saccharin, titanium dioxide sodium fluoride 0.24% (0.15% w/v fluoride ion) ‐ triclosan 0.30%

For randomization, the specimens (*n* = 72) were divided into six groups (*n* = 12) considering the proportional distribution of different color values (stratified randomization):

1. Immersion in distilled water without brushing (control group or D)
2. Immersion in distilled water and then brushing with Perfect White black toothpaste (DP)
3. Immersion in distilled water and then brushing with Colgate Total Whitening toothpaste (DT)
4. Immersion in cola (Coca‐Cola trademark regd., Khoshgovar Co., Tehran, Iran) without brushing (C)
5. Immersion in cola and then brushing with Perfect White black toothpaste (CP)
6. Immersion in cola and then brushing with Colgate Total Whitening toothpaste (CT)

The specimens were aged by thermocycling in a thermocycler (Vafaei Industrial, Tehran, Iran) for 5000 cycles between 5°C–55°C with a dwell time of 25 s and a transfer time of 10 s [7, 17]. After aging, the secondary color assessment of the specimens was performed using a spectrophotometer (*b*_2_, *a*_2_ *L*_2_). Then, the specimens in groups DP, DT, and D were immersed in distilled water, and the specimens in groups CP, CT, and C were immersed in carbonated cola beverage at 4°C for 6 days. The solutions were completely replaced after 24 h. According to a study, every 24 h of ceramic exposure to a beverage is equivalent to 30 days of exposure to the oral environment and beverage use [18]; therefore, to simulate 6 months, 6 days were required. The specimens were thoroughly washed with distilled water and dried with blotting paper.

Solutions containing a 1:1 weight ratio of distilled water to the desired toothpaste were prepared by mixing 5 g of the toothpaste with distilled water in an electric mixer for 30 s. The specimens in groups CP, CT, DP, and DT were brushed for 2.33 min at 40 rpm. At the end of each brushing interval, the ceramics were cleaned with 20 cc of distilled water for 20 s and completely dried with blotting paper. According to a study by Walsh et al. [16], the total brushing time was set at 120 s, twice a day, for all teeth. Because an individual has 28 teeth with 72 surfaces to be brushed, 240 s would be required to brush all 72 surfaces, which is 3.33 s per tooth surface per day, totaling 2.33 min of brushing over 6 weeks [16].

Then, the tertiary color of the specimens was determined spectrophotometrically (*b*_3_, *a*_3_, *L*_3_). Subsequently, *Δ* *E* values after aging compared with baseline (E2‐E1) and after brushing and immersion compared with before brushing (E3‐E2) were calculated using the following formula:

ΔE=l21−l2+a21−a2+b21−b2.

### 2.3. Statistical Analysis

The mean and standard deviation values were reported to describe the variables. Two‐way ANOVA was used to compare *Δ* *E* (E3‐E2) among the study groups (two variables: brushing and immersion). Considering the significant interaction effect of brushing and immersion (*p* = 0.07), subgroup analyses were performed by *t*‐test and one‐way ANOVA. As a secondary outcome, a paired *t*‐test was used to compare the effect of aging on color parameters. ANCOVA was used to compare color parameters after aging and immersion.

## 3. Results

### 3.1. Comparison of the Color Parameters After Immersion Compared With Baseline

The mean *Δ* *E*, *Δ* *L*, *Δ* *a*, and *Δ* *b* after immersion and brushing compared with baseline are listed separately for each group in Table 2.

**Table 2:** Mean *Δ* *E*, *Δ* *L*, *Δ* *a*, and *Δ* *b* of ceramic specimens after immersion and brushing compared with baseline.

Variable	Distilled water^a^	Distilled water and Colgate Total Whitening toothpaste^b^	Distilled water and Perfect White black^c^	Cola^d^	Cola and Colgate Total Whitening toothpaste^e^	Cola and Perfect White black^f^
*Δ* *L*	‐0.07 ± 0.31	0.10 ± 0.20	0.09 ± 0.30	0.11 ± 0.1^a^	0.13 ± 0.28	‐0.10 ± 0.17^a^
*Δ* *a*	‐0.09 ± 0.17	0.06 ± 0.14	‐0.01 ± 0.11	0.08 ± 0.08	0.19 ± 0.12	0.08 ± 0.10
*Δ* *b*	‐0.11 ± 0.33	0.16 ± 0.23	0.16 ± 0.26	0.16 ± 0.25	0.21 ± 0.20	0.16 ± 0.25
*Δ* *E*	0.44 ± 0.21	0.34 ± 0.17	0.43 ± 0.11	0.33 ± 0.08^b^	0.45 ± 0.09^bc^	0.32 ± 0.17^c^

^a^D: distilled water.

^b^DT: distilled water and Colgate Total Whitening toothpaste.

^c^DP: distilled water and Perfect White black.

^d^C: cola.

^e^CT: cola and Colgate Total Whitening toothpaste.

^f^CP: cola and Perfect White black.

*Δ* *E* : The highest mean *Δ* *E* was recorded in the CT, and the lowest in the CP group. The *Δ* *E* value in all groups was < 1, indicating it was not clinically detectable. The interaction effect of brushing and immersion was significant on *Δ* *E* (*p* = 0.02). Table 3 compares the effects of toothbrushing methods in distilled water and cola on *Δ* *E* and the effects of different solutions without toothbrushing, and with toothbrushing with Colgate Total whitening and Perfect white toothpastes on *Δ* *E*. No statistically significant differences were found between CT and C (*p* = 0.07), but the difference between groups CP and CT was significant (*p* = 0.05).

**Table 3:** Comparison of the effects of toothbrushing methods in distilled water and cola on *Δ* *E* and the effects of different solutions without toothbrushing, and with toothbrushing with Colgate Total Whitening and Perfect White black toothpastes on *Δ* *E*.

Solution	Toothbrushing comparison		Mean difference	95% CI	*p*value
D^a^	No toothbrushing 0.44 ± 0.21	T^b^0.34 ± 0.17	0.10	−0.08–0.27	0.37
		P^c^0.43 ± 0.11	0.01	−0.16–0.19	0.98
	T 0.34 ± 0.17	P 0.43 ± 0.11	−0.08	−0.25–0.09	0.46
C^d^	No toothbrushing 0.33 ± 0.08	T 0.45 ± 0.09	−0.12	−0.25–0.01	∗0.07
		P 0.32 ± 0.17	0.01	−0.12–0.15	0.98
	T 0.45 ± 0.09	P 0.32 ± 0.17	0.13	0–0.27	∗0.05
Group	*Δ* *E* in D	*Δ* *E* in C	Mean difference	95% CI	*p* value
No toothbrushing	0.44 ± 0.21	0.33 ± 0.08	0.11	−0.05–0.26	0.16
T	0.34 ± 0.17	0.45 ± 0.09	−0.11	−0.24–0.01	0.08
P	0.43 ± 0.11	0.32 ± 0.17	0.1	−0.03–0.23	0.11

^a^D: distilled water.

^b^T: tooth brushing with total.

^c^P: tooth brushing with Perfect White black.

^d^C: cola.

^∗^p < 0.05 indicates statistical significance.

*Δ* *L* : The *Δ* *L* parameter was negative for the CP (−0.1 ± 0.17) and D (−0.07 ± 0.31) groups, indicating that the color of these two groups had become darker, and it was positive for the other groups, which means that the color of the CT, C, DT, and DP groups had become lighter. The highest *Δ* *L* value was recorded in the CT (0.13 ± 0.28) group, and the lowest *Δ* *L* value was recorded in the CP (−0.1 ± 0.17) group. The *Δ* *L* value in all groups was < 1, which means that it was not clinically detectable. The interaction effect of brushing and immersion was not statistically significant on *Δ* *L* (*p* = 0.06).

*Δ* *a*: The *Δ* *a* parameter was negative for groups DP (−0.01 ± 0.02) and D (−0.01 ± 0.03), indicating that the color of these two groups shifted toward green, and it was positive for the other groups, which means that the color of groups CP, CT, C, and DT shifted toward red. The highest value of *Δ* *a* was recorded for the CT (0.12 ± 0/02) and the lowest for the D (−0.01 ± 0.03) group. The *Δ* *a* value for all groups was < 1, which means that it was not clinically detectable. The interaction effect of brushing and immersion was not significant on *Δ* *a* (*p* = 0.57).

*Δ* *b* : This parameter was negative for group D (−0.06 ± 0.02), which means that the color of this group shifted toward blue, and it was positive for other groups, which means that the color of groups CP, CT, C, DP, and DT shifted toward yellow. The highest value of *Δ* *b* was recorded in the CT (0.18 ± 0.05) group, and the lowest value was found in group D. The value of *Δ* *b* for all groups was < 1, meaning that it was not clinically detectable. The interaction effect of brushing and immersion was not significant on *Δ* *b* (*p* = 0.20).

### 3.2. Comparison of Color Parameters Before and After Aging

The *Δ* *E* value was 0.27 ± 0.55, which was not clinically detectable since it was below 3.3. The *Δ* *L* ^∗^ (mean difference: 0.20, *p* < 0.001), *Δ* *a* ^∗^ (mean difference: 0.19, *p* < 0.001), and *Δ* *b* ^∗^ (mean difference: 0.16, *p* < 0.001) all changed significantly, but they were all below 1 and were therefore not clinically detectable.

## 4. Discussion

This study investigated the effect of different whitening toothpastes on the color of aged ZLS ceramics after immersion in an acidic beverage. The use of charcoal and abrasive toothpastes and the use of cola did not have a significant effect on *Δ* *a* and *Δ* *b* parameters. The color of all specimens shifted toward yellow based on the CIE L ^∗^a ^∗^b ^∗^ color space, except for the D group (immersion in distilled water without brushing). Also, the color of all groups shifted toward red, except for the D and DP groups, which shifted toward the green axis. The two toothpastes showed similar behavior in *Δ* *L* in distilled water and cola, with no significant difference.

Cola consumption and simultaneous brushing with charcoal toothpaste resulted in darkening of the specimens, although the discoloration was minimal. Therefore, if a person consumes cola and intends to use a whitening toothpaste, based on the results of the present study, Colgate Total Whitening toothpaste may significantly brighten his teeth compared with charcoal toothpaste and yield a whiteness comparable to the control group. The presence of titanium oxide, which is a white pigment, in the composition of Colgate Total Whitening toothpaste may have caused precipitation of this pigment and lightening of the specimens. Also, the acidic pH of cola and the presence of limonene acidic compound in the composition of Perfect White black toothpaste may have had a synergistic effect, causing dissolution of the ZLS ceramic surface, which may have caused surface roughness and precipitation of more black compounds of the Perfect White black toothpaste on the surface of specimens, resulting in their darkening.

In immersion in cola and distilled water, the pattern of *Δ* *E* for comparing the two types of toothpastes showed that only with the simultaneous use of CT, the *Δ* *E* was significant, meaning that the use of CT caused greater *Δ* *E* in the specimens than the use of CP. However, this difference was not clinically detectable. Numerically, all specimens showed *Δ* *E* values below 1, which are not visually detectable by laypeople or professionals [19, 20]. *Δ* *E* below 3.3 is not clinically detectable, and no specimen was observed with a *Δ* *E* value higher than this threshold in the present study. Both types of toothpaste showed similar *Δ* *E* values in distilled water and cola.

A search of the literature did not reveal any study on the simultaneous effects of beverage consumption and toothbrushing on ZLS ceramic color parameters. Most studies that evaluated the optical properties of ZLS under various interventional conditions such as thermocycling [11, 12, 21, 22], ultraviolet light cycling [23], gastric acid solution [14], coloring solutions [24, 25], and coloring solution plus thermocycling [17] measured different optical properties such as *Δ* *E* [12, 17, 21, 23, 25], translucency parameter [11, 12, 17, 21, 23, 25], contrast ratio [17, 23], and opalescence [11, 21]. Therefore, a direct comparison of the present study′s results with those of these studies is not possible.

Different mean *Δ* *E*_*a* *b*_ values of 0.6 [21], 3.78 [17], 4.56 [24], and 5.21 [25] have been reported in the literature for ZLS ceramics postintervention. The present results are consistent with the study that reported the minimum *Δ* *E* [25]. Another study reported a *Δ* *E* value of 3.78 [17] following immersion of ceramic specimens in orange juice and simultaneous thermocycling for 5000 cycles. Studies that reported *Δ* *E* values of 4.56 [24] and 5.21 [17] used coffee solution. In another study [24], the specimens were immersed in coffee for 2 months, equivalent to 5 years of coffee consumption, whereas another study [17] used 1 month of immersion, equivalent to 2.5 years of coffee consumption. Since the color of coffee beans is polar, it penetrates deep into the specimens [26], and may yield a larger *Δ* *E* value. Studies that used acidic and coloring solutions to investigate the optical properties of ZLS ceramics used coffee [17, 24, 25], orange juice [17], Coca‐Cola [17], tea [17, 24], and gastric acid [14], and the pH of these solutions varies greatly, with coffee pH being 6.9, Coca‐Cola being 2.5, tea being 4.9, orange juice being 3.5, and gastric acid being 1.2. These different pH values have resulted in different surface behaviors of ceramics [14, 17]. In the present study, cola was used since it is a widely consumed drink with high erosive potential due to its low pH, which is within the range of most acidic beverages [27]. Cola includes phosphoric acid, carbonated water, sugar, and caramel color [28, 29]. Caramel pigments are less polar than coffee pigments, which prevents them from penetrating deeply into the substrate structure. Thus, it mainly remains on the substrate surface as an external pigment [30]. It was probably removed from the surface by whitening and charcoal toothpastes. On the other hand, the 6‐month consumption period considered in this study may not have been sufficient to show the effects of cola drinking. In the present study, two types of whitening toothpastes were used: one with an abrasive mechanism and the other containing activated charcoal. It appears that because of the high porosity and large surface area provided by charcoal particles (1000 mL/g), these particles can absorb surface pigments and stains, which are later removed by brushing the pigments absorbed by the charcoal, and the tooth surface is cleaned as such [31, 32].

The presence of two types of surfactants, sodium lauryl sulfate and cocamidopropyl betaine, in the composition of charcoal toothpaste causes greater dispersion of this toothpaste in the oral cavity. On the other hand, since this toothpaste contains phosphate anticalculus compounds with an unpleasant taste, the lemon‐flavored limonene has been added to it, which has low acidity [32]. With these observations, it was perhaps expected that this toothpaste would cause obvious changes in the color of the specimens, but no such result was obtained in this study. The Colgate Total Whitening toothpaste contains titanium oxide pigments, which serve as opacifiers [6]. This compound can be deposited on the ceramic surface and improve the lightness of ZLS specimens immersed in cola.

Regarding the reduction in lightness of the CP group, it can be stated that the low pH of cola, along with the presence of limonene in the composition of Perfect charcoal whitening toothpaste, which has an acidic pH, damaged the ceramic surface. The charcoal, pigment, and caramel particles were not completely removed from the specimen surface by brushing, creating a rough surface that resulted in greater penetration depth of the caramel and charcoal pigments. Regarding the effect of Perfect charcoal whitening toothpaste on the properties of other CAD/CAM ceramics, a study conducted on 3 types of CAD ceramics namely feldspathic, zirconia, and lithium disilicate ceramics stated that 3 years of use of Perfect charcoal whitening toothpaste caused discoloration in the ceramic specimen; after 5 years of use, severe discoloration was observed, whereas within 6 months of use, only the surface gloss of the ceramics was lost [15]. Accordingly, 6 weeks of use in the present study may be too short to notice a significant change.

One limitation of this study was its in vitro design, which does not reflect the dynamic conditions of the oral cavity and the role of influential factors such as saliva and its buffering capacity, pH, dilution of acidic drinks in the oral cavity, thermal alterations, and occlusion. The smooth surface of the specimens, compared with the three‐dimensional shape of the teeth in terms of convexity and contour, makes the conditions differ from those of the oral environment, which might have affected the results. On the other hand, the period considered for aging and the use of toothpaste and drinks in this study was short. Future studies are required to simulate longer consumption times for carbonated drinks and toothpaste, use a longer aging period, and consider the abrasive effects of whitening toothpastes. Further studies to evaluate the effect of these materials on the surface roughness are recommended.

Furthermore, the surface topography and roughness of the specimens should be evaluated in future studies, preferably using microscopy, to obtain more accurate results. Also, further studies should tend to evaluate the results considering color change more subjectively, as the objective results may have no clinical or observational significance.

## 5. Conclusion

Within the limitations of this in vitro study, the results showed that using Perfect White black and Colgate Total Whitening toothpastes for 6 weeks and consumption of cola for 6 months did not cause a clinically significant color change visible to the naked eye in aged ZLS ceramic specimens. Use of charcoal toothpaste for 6 weeks, with 6 months of cola consumption, resulted in darkening of aged ZLS ceramic specimens. In contrast, the same conditions with Colgate Total Whitening toothpaste resulted in improved lightness, neither of which was clinically detectable. Use of charcoal and abrasive whitening toothpastes alone did not cause changes in the lightness of aged ZLS ceramic specimens.

## Funding

This study was supported by the Dentistry Research Institute of Tehran University of Medical Sciences, 99‐3‐134‐51472.

## Conflicts of Interest

The authors have nothing to report.

## Data Availability

The data supporting the findings of this study are available upon request from the corresponding author. The data are not publicly available due to privacy or ethical restrictions.
